# Evidence of geoelectrical resistivity values on groundwater conditions in Wadi El Natrun and its vicinities, West Delta, Egypt (cases studies)

**DOI:** 10.1038/s41598-022-12644-0

**Published:** 2022-06-24

**Authors:** Fardous M. Zarif, Ahmed M. Elshenawy, Mostafa S. M. Barseem, Abdalla A. Al-Abaseiry, Ahmed N. El Sayed

**Affiliations:** Geophysical Exploration Department, Desert Research Center, Matariya, Cairo, Egypt

**Keywords:** Environmental sciences, Hydrology

## Abstract

Recently, Wadi El Natrun and its surroundings have witnessed intensive investments in land reclamation, including the arbitrary drilling of hundreds of groundwater wells. Currently, serious hydrogeological and environmental problems have been addressed, such as groundwater quality degradation and water head drop. Electrical resistivity measurements were performed at six locations across the study area to assess its ability to reveal the heterogeneous subsurface stratigraphic and hydrogeological setting of groundwater aquifer(s). The geoelectrical results successfully reflect the current vulnerable hydrogeological setting of the study sites. The current study highlights the current practice in which farmers rely on isolated 1-dimensional vertical electrical sounding (1D VES), which is not the only exploration tool for such electrically conductive stratigraphic succession. One of the main findings is addressing the advantage of applying 2-dimensional electrical resistivity imaging (2D ERI), where it offers a more robust view of both vertical and lateral variation of the investigated subsurface section (Case 3). On the other hand, the Geographic Information System (GIS) could mirror the present groundwater potentiality status, where both GIS analysis and resistivity results coincide, and where the good potentiality zone is restricted to the west and southwest directions of the study area (area of interest (aoi)), where the resistivity values of water bearing are relatively high and lie on the main drainage (Cases 2, 5, and 6). On the contrary, poor potentiality zones are deemed because of their proximity to tiny attributers, and are characterized by low resistivity values (Cases 1, 3 & 4), Finally, the current research study demonstrates the significance of combining morphometrical analysis with geophysics techniques for such environmental problems, where groundwater is primarily controlled by geomorphological features and geological conditions, including lithology and geological structures.

## Introduction

Many state plans are focused on transforming the wadi El Natrun into agricultural, industrial, and residential centers for the benefit of the densely populated Nile Delta. Groundwater is considered to be the main water resource for any of such activities for developments. In the last two decades, serious hydrogeological and environmental problems have been raised^1,2^. El Natrun and Nubariya were founded as a result of the rapid expansion of reclaimed lands and inhabited area. The scarcity of fresh water, combined with a large reduction in groundwater levels, has raised demand for groundwater, exposing water resources to threat in both type and amount^1,3–5^. The aim of this research is to assess the potential of geoelectrical investigations to expose the diverse subsurface stratigraphic and hydrogeological settings of groundwater aquifers (s) in various case studies throughout the study region. 1D VES is the most often used approach in most groundwater research, as detailed in many publications^6–10^, where the resistivity method is particularly sensitive to subsurface structure exchange. Several research projects have been conducted in Wadi El Natrun and its environs, but groundwater conditions still require more investigation.

A Geographic Information System (GIS) also allows for the efficient management and integration of massive volumes of geographical and temporal data. This will help to narrow down the promising zones for additional hydrogeological and geophysical studies on the ground, ultimately finding the promising drilling sites. As a result, the incorporation of GIS serves as a critical tool in the successful regional evaluation of groundwater pollution zones^11^. Furthermore, in a place with difficult geology, such as Wadi El Natrun, using traditional electrical resistivity techniques in groundwater delineation is crucial because it assists in the identification of unseen subsurface hydrogeological heterogeneities.

## Materials and methods

### Site location and geomorphologic, geologic and hydrogeologic setting

**Figure 1:**
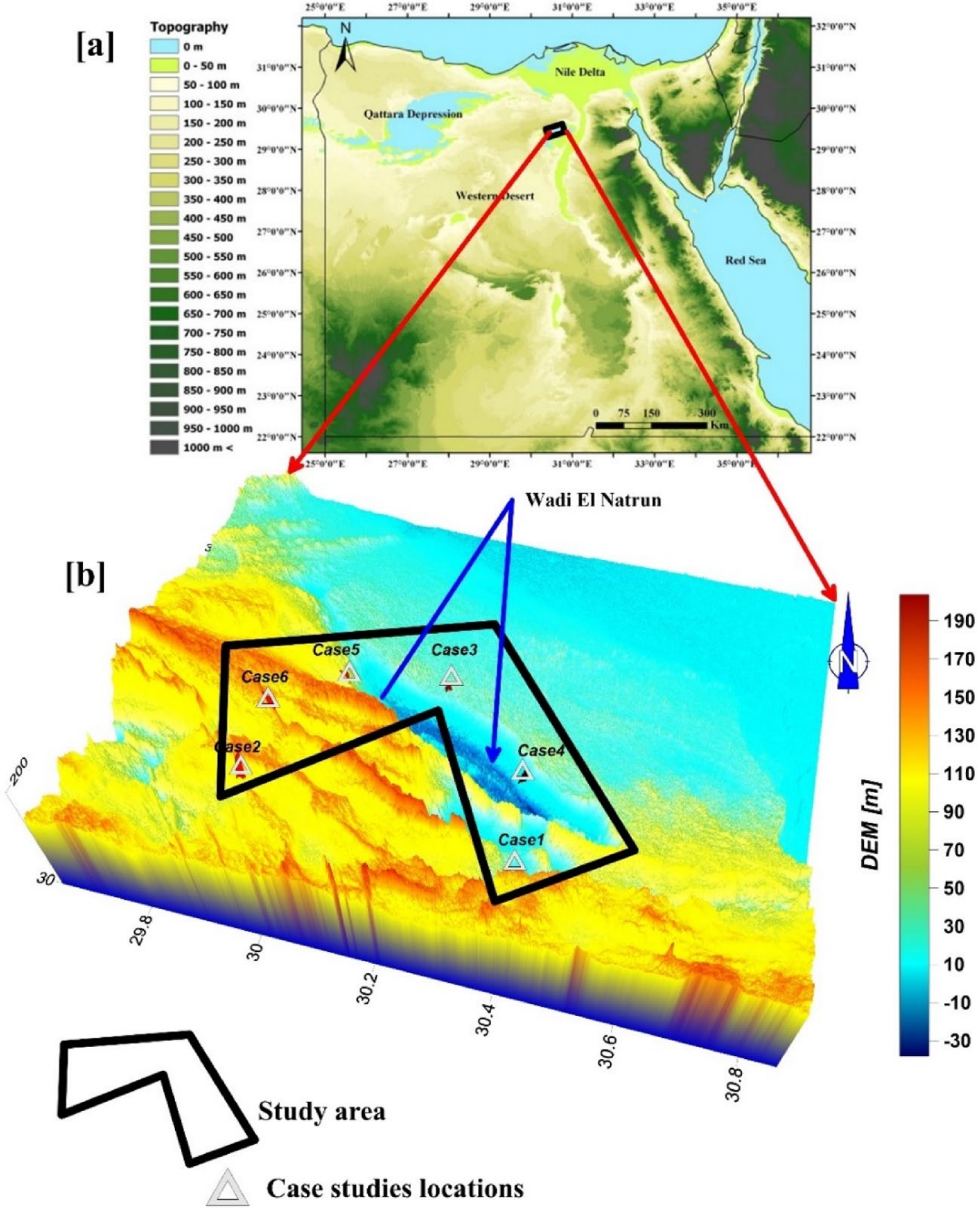
(**a**) Topographic map of Egypt containing the study area. (**b**) Location map of the study area with six case studies.

The cases being studied are located to the east, west, and southwest of Wadi El Natrun (Fig. 1). Longitudes 29.80 and 30.60 E, and latitudes 30.20 and 30.80 N, form its boundaries. The current study focuses on the geological effects on water-bearing structures in the west Nile Delta area. It is located in Egypt's extremely dry area and is distinguished by a hot, lengthy summer and a short, mild winter. Maximum and lowest air temperatures are typically recorded in August (35.7 °C–21.9 °C) and January (19.4 °C–7.8 °C). The average yearly rainfall intensity steadily rises northward. It averages around 50 mm/year in the northern half of Wadi El Natrun and reaches 190 mm/year towards the Mediterranean coast^12^.

**Figure 2:**
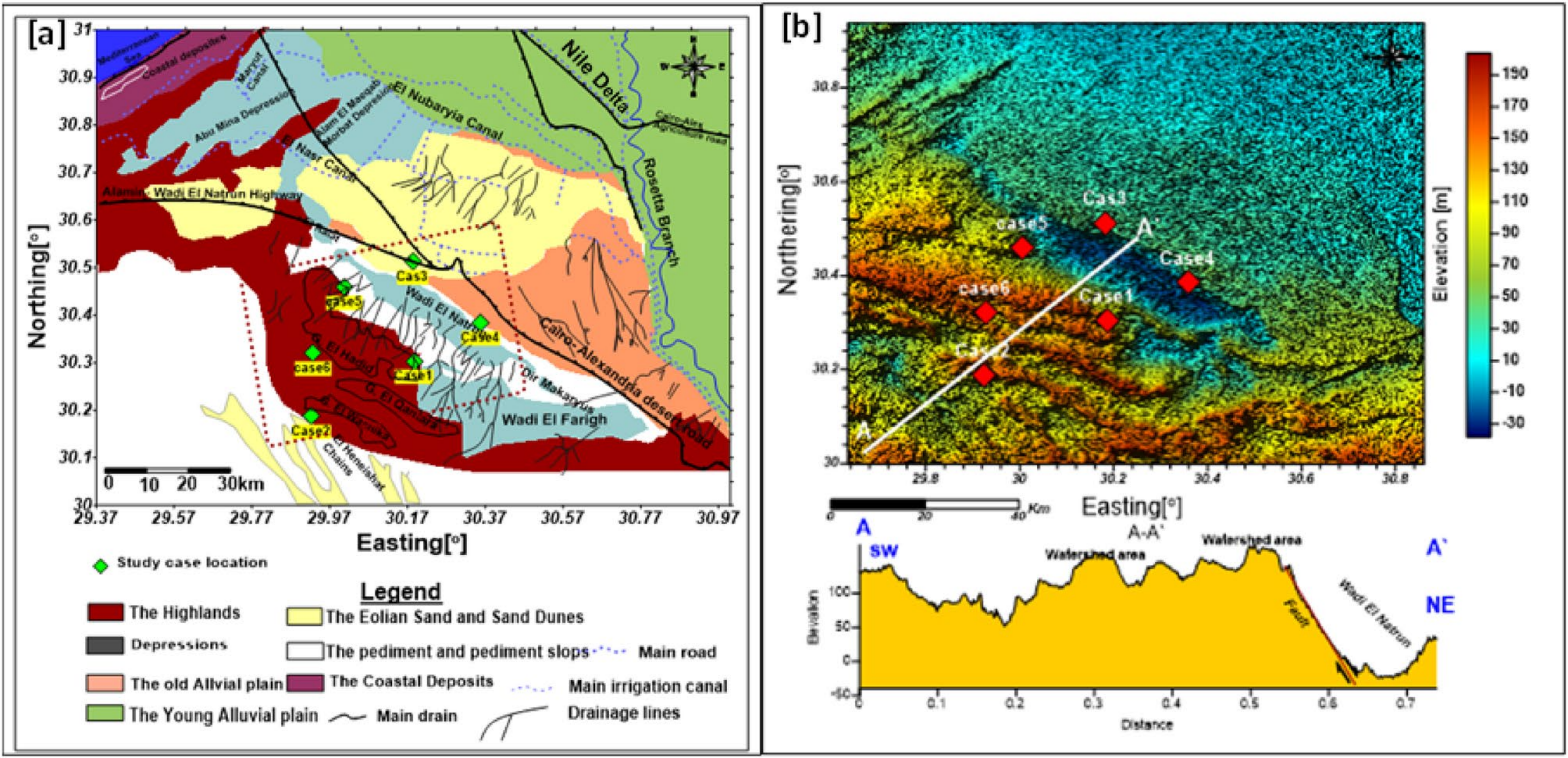
(**a**) Geomorphologic map of West Nile Delta and Wadi El Natrun. Green symbols show case studies locations. (**b**) Digital Elevation Image map with profile A–A′ crossing the study cases showing main geomorphic unit. Red symbols show locations of case studies.

Geomorphologically, according to^13–18^, some topography forms in the western part of the Nile Delta suggest that aridity prevailed recently (e.g., drift sand accumulations, salt lakes, and marches). Other landforms show that conditions were less arid during the Late Tertiary–Early Pleistocene epoch (e.g., old dry drainage lines and immense depressions). They said that the area west of the Nile Delta is commonly defined by the following geomorphic units, from north to south (Fig. 2). According to^10^, the study area consists of four main landforms—the coastal plain, structural plain, tablelands and sandy plain. The depressions constitute a main land feature that influences the landscape of the present area. Wadi El Natrun (− 23 m) and Wadi El Farigh (− 4 m) depressions exhibit various morphologic features due to the influence of the local geologic and topographic conditions^6^. The study area has low relief and gentle terrain, with elevations ranging from below sea level (− 30 m) to more than 190 m (Fig. 2b). Generally, the investigated area slopes gently towards the northern and eastern directions.

**Figure 3:**
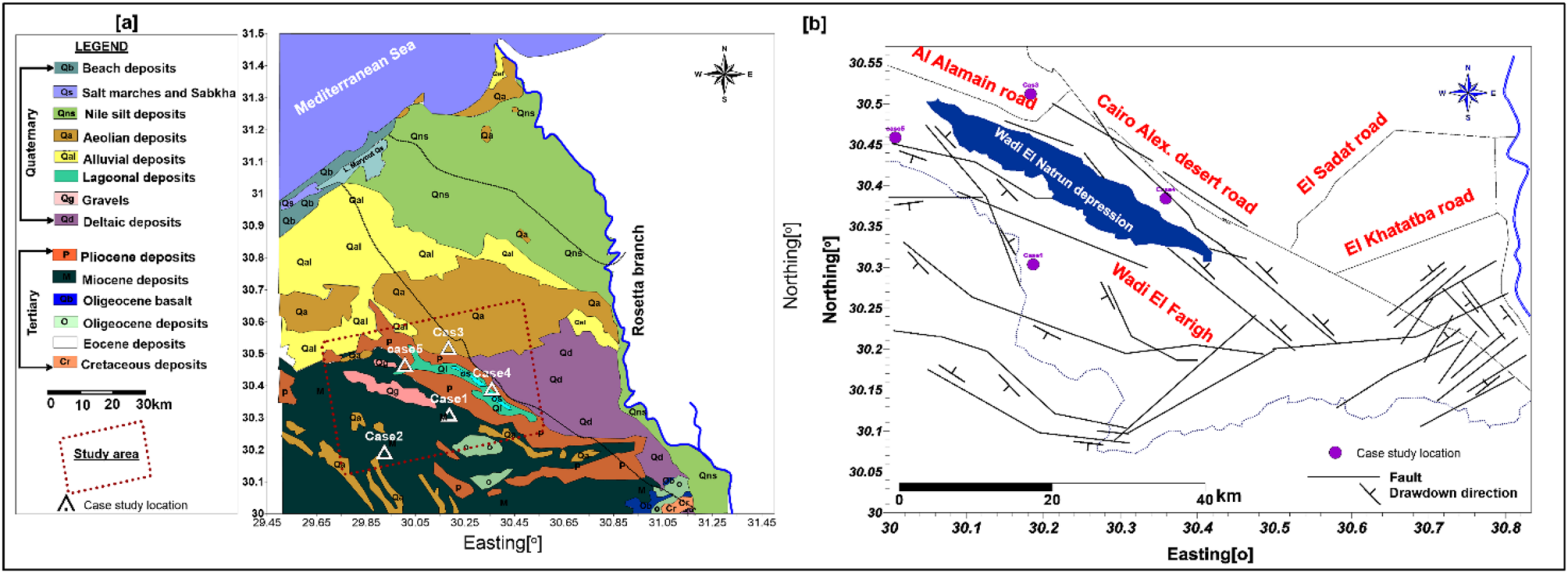
(**a**) Geological map of the study area (after ^19^), (**b**) Structural map of the study area (after ^20^).

Geologically, the study area (Fig. 3a) has been discussed by several authors, e.g.^13–18^. Generally, the region west of the Nile Delta is covered by extensive exposures of sedimentary successions belonging to Tertiary and Quaternary ages. The southern and western portions of Wadi El Natrun depression are dominated by Tertiary sediments (Pliocene and Miocene). They are characterized by sand and sandstone with clay and limestone intercalations. Pliocene sediments have a wide distribution in the Wadi El Natrun depression and the adjacent areas.

Due to Said^12^, El Moghra formation belongs to the Early Miocene and it is composed of sand, sandstone and clay interbeds with vertebrate remains and silicified wood. This formation is represented south and west of Wadi El Natrun. The Quaternary deposits cover wide stretches of the area and are detected in water wells and it distinguished into different types such as aeolian sands, lagoonal deposits, deltaic deposits, and crust. The aeolian sands are found in the lowland area of the big depressions of Wadi El-Natrun. To understand how some distant points may be clustered together, it was reported that the Pliocene sediments were affected by several (NW–SE) faults which bound it in the east with a downthrown side towards the north east^21^. Figure 3b as well, the depression was bounded from the south and west by Clysmic faults (NW–SE) with downthrown side to the northeast that facilitates the hydraulic linking between the Pliocene and Delta Aquifer^22^. These faults connect the Pliocene with the Miocene aquifer south enhancing groundwater flow from the SE to NW^18,22,20^.

El Sheikh^15,23^ categorized the aquifers of Wadi El Natrun hydrogeologically into three distinct waterbearing formations to the west and north of Wadi El Natrun. These are the Pleistocene, Pliocene, and Miocene aquifers^24,25^. The Pleistocene aquifer is located between the Nile's Rosetta branch and the eastern parts of wadi El Natrun, which slopes gradually eastward and northward. The main aquifer is composed of Pleistocene layers of Nile sands and gravels with thin bands of clay. The aquifer thickness begins at about 150 m near Wadi El Natrun and gradually increases eastward until it reaches about 300 m. The Pleistocene aquifer's groundwater is largely under a free water table and/or semi-confined condition. The groundwater quality of the Pleistocene aquifer varies between fresh and slightly brackish^22,26,27^.

A shallow Pliocene aquifer is observed in the north due to seepage from surface water canals which deepen southward to reach about 40 m near the border of Wadi El Natrun. Its maximum thickness is about 140 m, while the penetrated thickness varies between 100 and 130 m. The sediments are widely distributed in a number of localities and are mostly built up of clay underlying the Pleistocene aquifer^15^. The quality of the Pliocene groundwater ranges generally between fresh and slightly brackish water^1^.

Moghra formation of Early Miocene sediments has excellent occurrences in a number of locations, including Gebel El Hadid, Dahr El Tashash, Khashm El Kaoud, and northwest of Wadi El Farigh. It is made up of sand, sandstone, and clay lenses, and serves as an excellent aquifer, and various water wells have been dug in it. The deep water of the Miocene aquifer is accounted for by this high structure; the water level is sloping westward^28^. The quality of the Miocene groundwater varies by region. It is normally fresh in the southern regions, where direct recharge comes from the Nile Delta's west, and reasonably fresh to brackish in Wadi El Natrun and its western parts^29^.

A recent study by^1^ (2020) involved That the majority of water well samples in Wadi El Natrun and surroundings fall into one of two categories (Na–Cl–HCO3 and Na–Cl–SO4). The water type changes with distance from the depression axis: when one gets closer to the depression, the water changes to the Na–Cl–SO4 type. Salinization (Na–Cl–SO4 rich) may have occurred locally as a result of the depression's mixing of paleowater and Nile Delta aquifer water. Evaporation, salt dissolving or seawater intrusion, and salinization of groundwater resources have been thought to have a significant impact and, when combined with high levels of abstraction, have been thought to be the direct cause of water quality change.

### Methods

**Figure 4:**
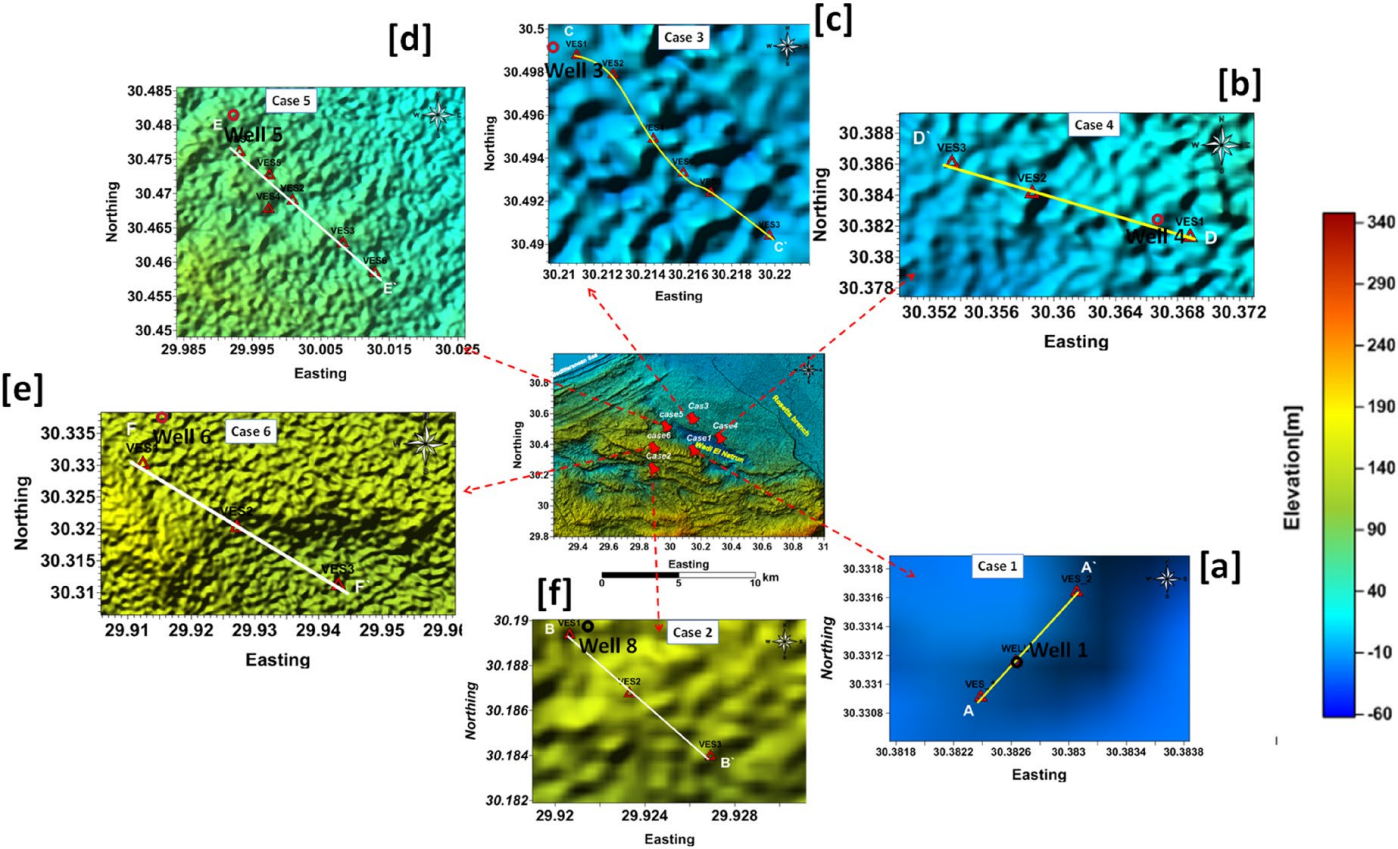
Location map of the case studies at wadi El Natrun and its vicinities.

The Schlumberger array setup was utilized to acquire 18 VES measurements. This array's configuration has been shown in several publications, including^11,30^. The maximum current electrode (AB) spacing ranging from 1000 to 1500 m. Terameter SAS 1000 was used to measure the apparent resistivity of the subsurface layers. The collected data was utilized to evaluate the electrical characteristics of subsurface models (thicknesses and real resistivities)^7^. To develop the first model and calibrate the acquired data, a number of available wells from the analysed instances are employed. The measured VES were inverted using the Programme IX1D, which Interpex Limited patented in 2001 and 2002^31^. The inverted data was represented in geoelectrical cross sections and contour maps. 1D VE soundings were conducted at six distinct places throughout the Wadi El Natrun depression to better understand the underlying architecture and groundwater conditions in such situations. In the first scenario, two VESes are performed near to the drilling well (1), and the cross-section A-A' is built going NE-SW (Fig. 4a).

Cases 2 and 6 are located in the western and southern portions of the Wadi El Natrun basin, respectively. 3 VE soundings are performed and joined together in B–B′ and F-F' cross-sections that run NW–SE and pass via wells (8) and (6), respectively (Fig. 4e,f). In these cases, the maximum current electrode spacing is 1500 m to reach a depth of roughly 150–180 m (Fig. 4). In the third and fifth cases, 6 VE soundings were performed along the built cross sections C–C′ and E–E′, as illustrated in Fig. 4c,d. Cases 3 and 5's interpretation data were calibrated using Wells 3 and 5. Furthermore, because of the recorded lateral facies change, one (2D ERI) was done, specifically on case 3, to complement the picture of the subsurface structure of this case. The Syscal Junior equipment is used to conduct a 2D ER imaging profile in the NW–SE direction with 10 m interval spacing between 72 electrodes using wenner array in NW–SE direction. According to^18,32,33^, "there are many reasonable reasons to employ 2D ERI, which is frequently used for hydrogeological investigations and environmental challenges." 2D ERI measurements were inverted using L1-norm inversion using Res2dinv.

The GIS approach is based on the advanced space-borne thermal emission and reflection radiometer (ASTER), which generates a digital elevation model (DEM) with a spatial resolution of + 30 m and was utilized in this work to assess surface runoff recharging and movement. This approach is commonly used to generate runoff potential maps for small to medium-sized involved drainage basins. The drainage network, according to Mark^29^, is defined as the sites at which surface runoff is linked and concentrated by slope mechanisms. There are several ways to obtain drainage networks, including topographic maps, aerial photography, field studies, satellite imagery, and, more recently, digital elevation models. ArcGIS v10.4.1 was used to apply hydrology tools to handle the fill of all sinks in the resultant mosaic raster. DEM analysis was used to determine flow direction, basins, stream orders, slopes, and shaded relief^34^.

## Results and discussion

The resistivity method was chosen in this study due to its sensitivity to water salinity and clay content which plays an important role in predicting the groundwater potentialities as resistivity decreases when the salinity increases. As many authors reported in their publications that if clays are present in the soil, the value of the resistivity decreases^33,35,36^.

### Case 1

**Figure 5:**
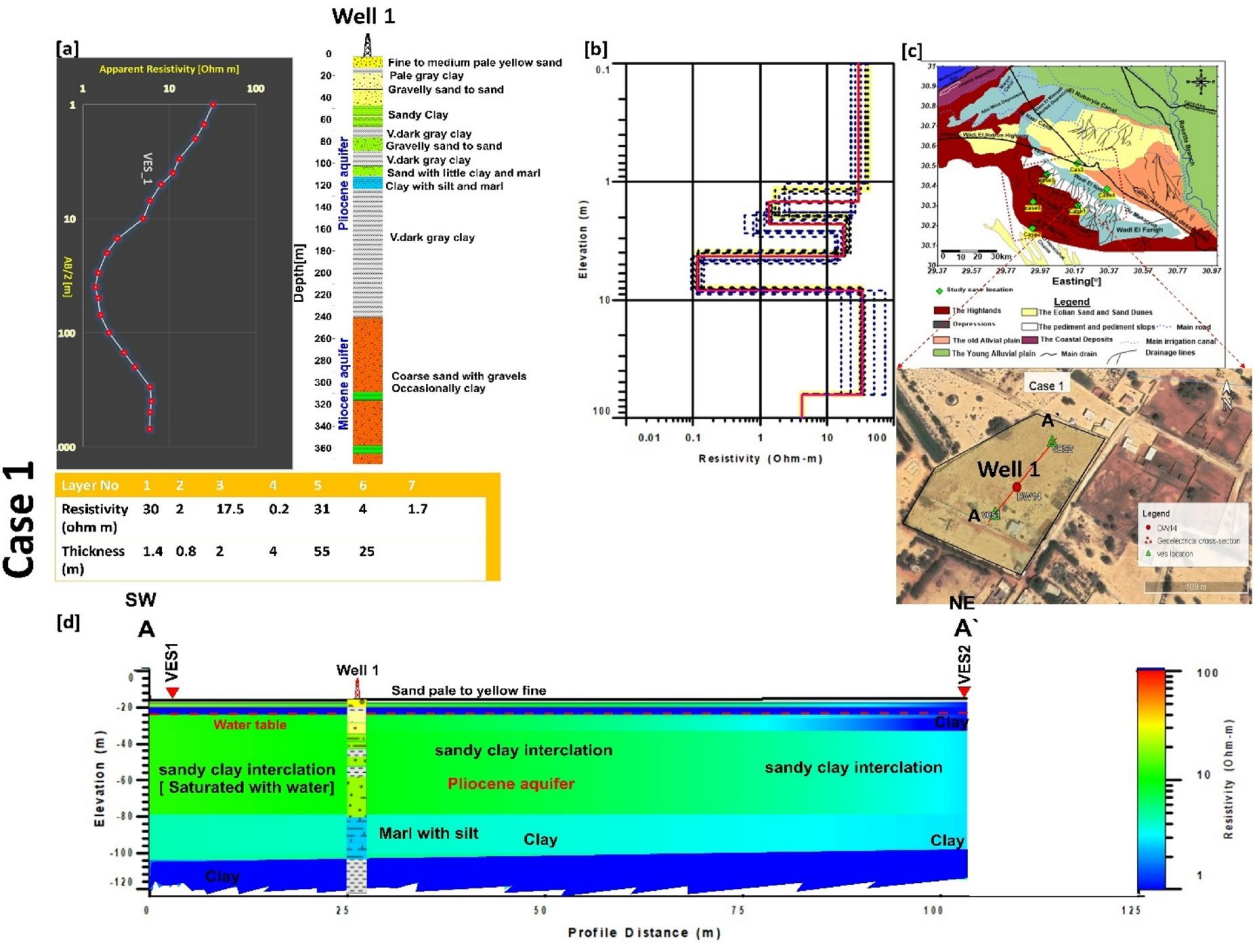
(**a**) VES1 as example for case 1 with Well_ 1, (**b**) inverted model with cumulative models, (**c**) the location of A–A′ cross section in the study area. (**d**) inverted resistivity cross section AA′ in the first study area.

This case is located east wadi El Natrun particularly in Bani Salama area (Fig. 5). Drilling has revealed that the majority of the succession is formed of clay and sandy clay, which has an influence on the resistivity values of the water. The water level is approximately 15 m below the ground surface, and the salinity is greater than 5000 ppm. The first model for data calibration was created using drilling data of Well 1. Five geoelectrical layers were resulted from the inverted two VESes along A-A′ cross section. The resistivity values in the first layer range from 20 to 50 Ωm, and the relative changes are connected to the sorting of the grain size of the sand and clay content, which reduces the resistivity values. The thickness ranges between 1.5 and 2.5 m. The next four layers are recorded as partially saturated to fully saturated with water with intercalation clay interbed (well drilling information), whereas the second and fourth layers are recorded as low resistivity layers of varying thickness. At VES 1, the second layer has resistivity values ranging from 5 to 10 Ωm with a thickness of 2–5 m, while the fourth layer has resistivity values less than 0.5 m with a thickness of 15 m (around 28 m depth). These strata are made up of clay and marl that has been saturated with saltwater water (high clay content). The third layer, on the other hand, was found to have a low resistivity (10–20 Ωm) and thickness (15–25 m). This layer is confirmed by well 1, which is made up of fine sand and silt and has a water level of 5–8 m. The last layer is found at a depth of 100–150 m and has low resistivity values observed (5–10 Ωm). The high clay content within the fine sand layers is responsible for the relatively low resistivity in that section of Wadi El Natrun and, as a result, the restriction of groundwater potential (Fig. 5).

### Case 2

**Figure 6:**
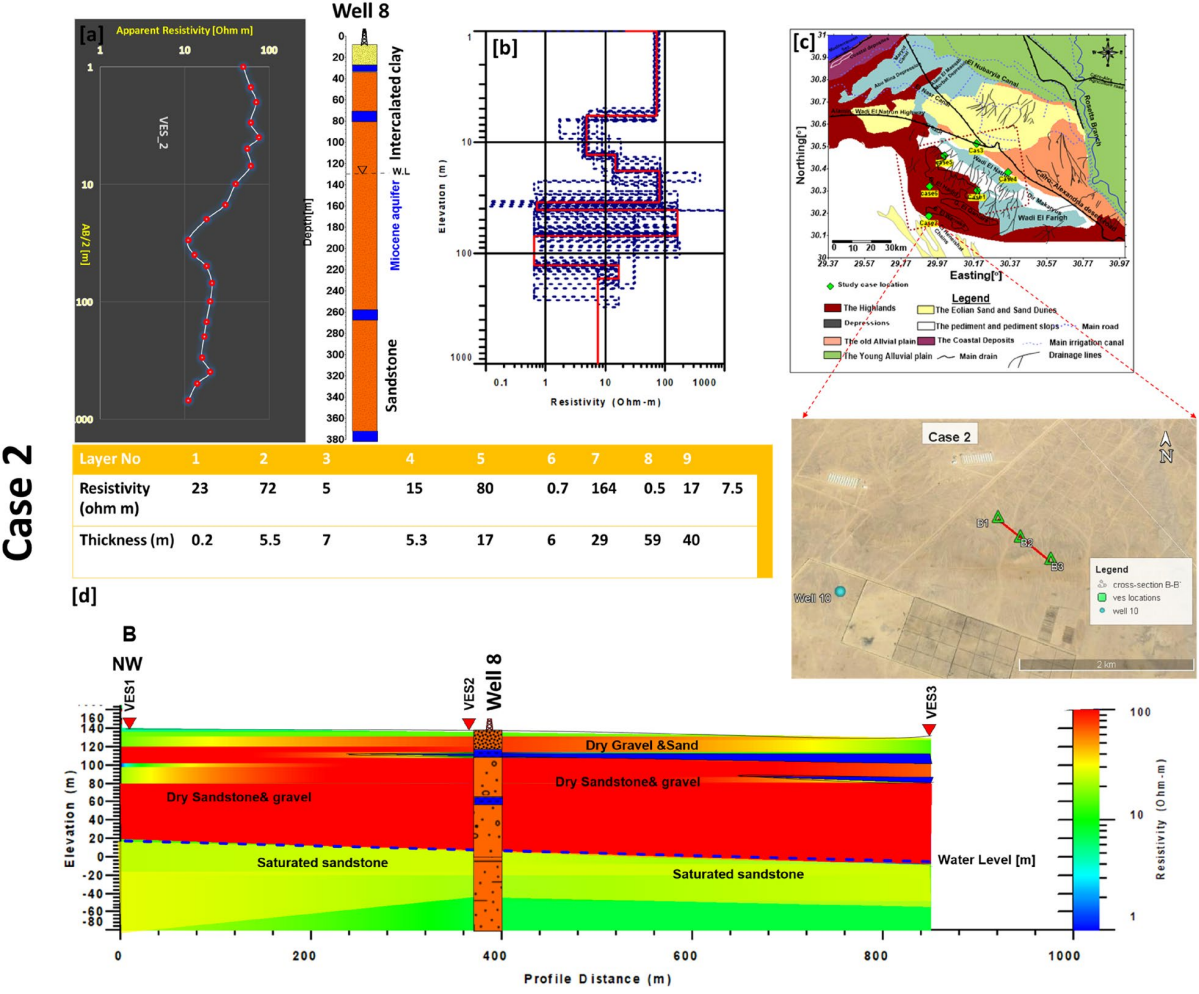
(**a**) VES2 as example for case 2 with Well 8, (**b**) inverted model with cumulative models, (**c**) the location of BB` cross section in the study area, (**d**) inverted resistivity cross section BB′ in the Second study area.

It is located in the south-west of Wadi El Natrun. The water level exists at a depth range from 120 to 160 m, and the salinity is > 2700 ppm. Well 8 was used to create the initial model for data calibration. The results of the interpretation are four geoelectrical layers along B–B′ cross section (Fig. 6). The first layer is composed of three dry sublayers of sand and gravel with resistivity values of 50–567 Ωm and its thickness varies between 5 and 35 m. The second layer is also dry and has resistivity values varying between 60 to 720 Ωm, but its thickness is recorded at 55–120 m. Both layers began at a depth of 35 m and ended at a depth of 120–160 m, and they are considered the unsaturated zone that overlies the Miocene aquifer at this location. The third layer is representing the saturated zone (water bearing layer) where its resistivity is less than 50 Ωm and its thickness ranges between 50 and 65 m, and its depth varies from 170 to 235 m. The last layer (fourth layer) is considered a layer of clay or clayey sand due to the low resistivity value (< 15 Ωm).

### Case 3

**Figure 7:**
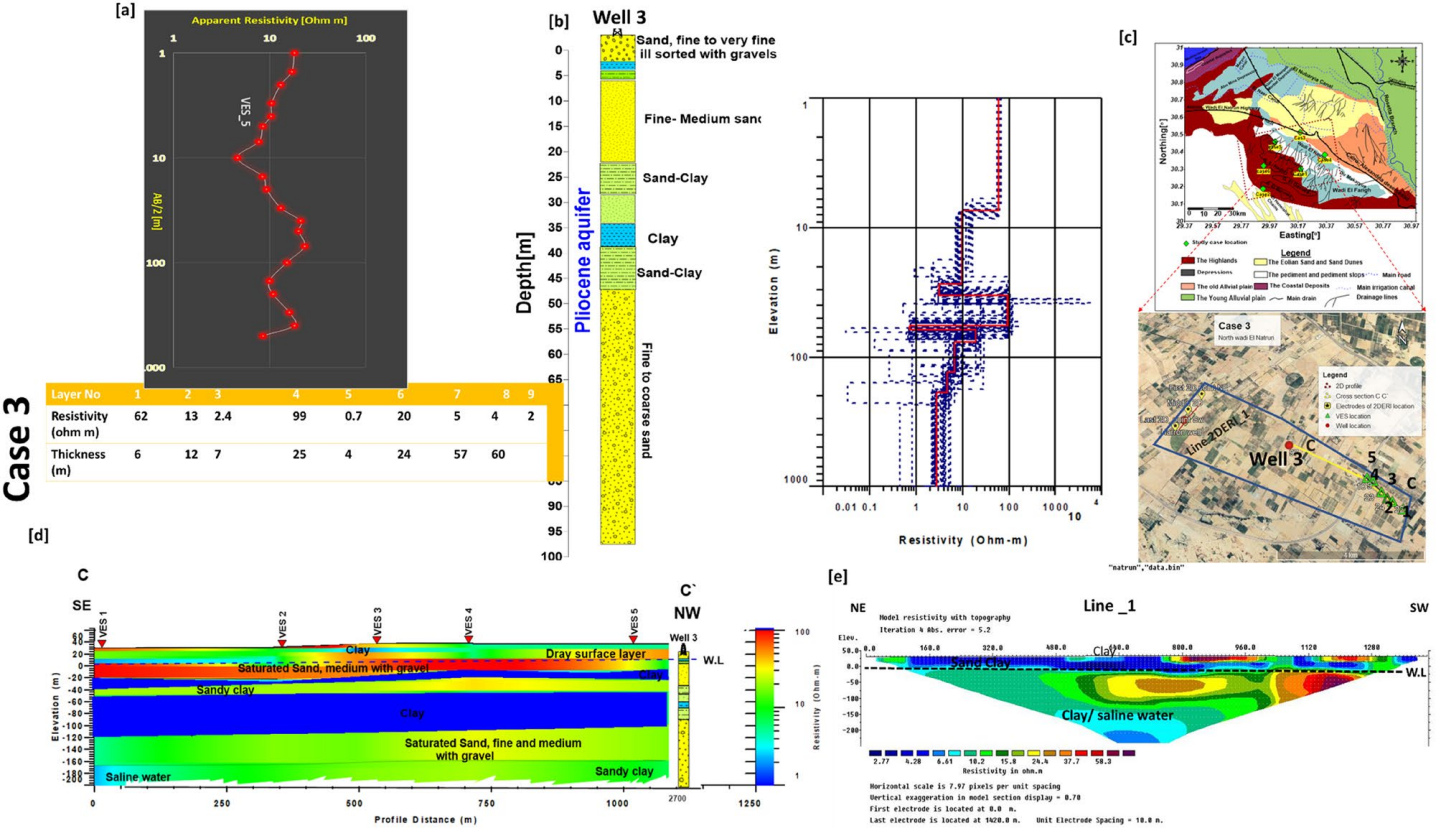
(**a**) VES1 as example for case 3 with Well 3, (**b**) inverted model with cumulative models, (**c**) the location of C–C′ cross section in the study area, (**d**) inverted resistivity cross section CC′ in the third study area. (**e**) Line_1 of 2DERI in the opposite direction of C–C′ cross-section of VES.

This case is located to the northwest of Wadi El Natrun. The fundamental issue in this area is that groundwater wells are suffering from increasing water salinity over time, which has a significant influence on the quality and quantity of the ensuing crop. The water level is between 33 and 35 m deep. The salinity of groundwater increased from 900 ppm in 2014 to 2700 ppm in 2020^2^. The original model for data calibration of 1D and 2D electrical resistivity imaging was created using Well 3. C–C′ cross-section was created by assembling five VESes (v1, v2, v3, v4, v5) in the SE-NW direction (Fig. 7). In contrast, 2D ERI of profile 1 was performed in the crossing direction (NE-SW) to follow the facies changes.

The findings of both the 1D and 2D ER interpretations revealed the presence of five geoelectrical layers. The first layer has resistivity values ranging from 50 to 100 m and fluctuates between 1.5 and 33 m, and it is followed by a layer with quite high resistivity values ranging from 130 to 150 m. This layer of saturated sandstone with fresh water has a thickness of 15–20 m, where the third layer has a 20 m saturated layer of sandy clay at a depth of 60–65 m, separated by a 5 m clay layer. The saturated sand layer has an interbedded clay lens, lowering the resistivity to < 5 Ωm. An extremely low resistivity layer of 1 Ωm with a thickness of 50 m at a depth of 80 m was observed by the interpretation. Due to available well drilling information, this layer is mainly clay. Due to the reported low resistivity value (5Ωm), the fifth layer recorded was a saturated layer with salty water. This layer was found at a depth of 150 m and has a thickness of more than 60 m.

### Case 4

**Figure 8:**
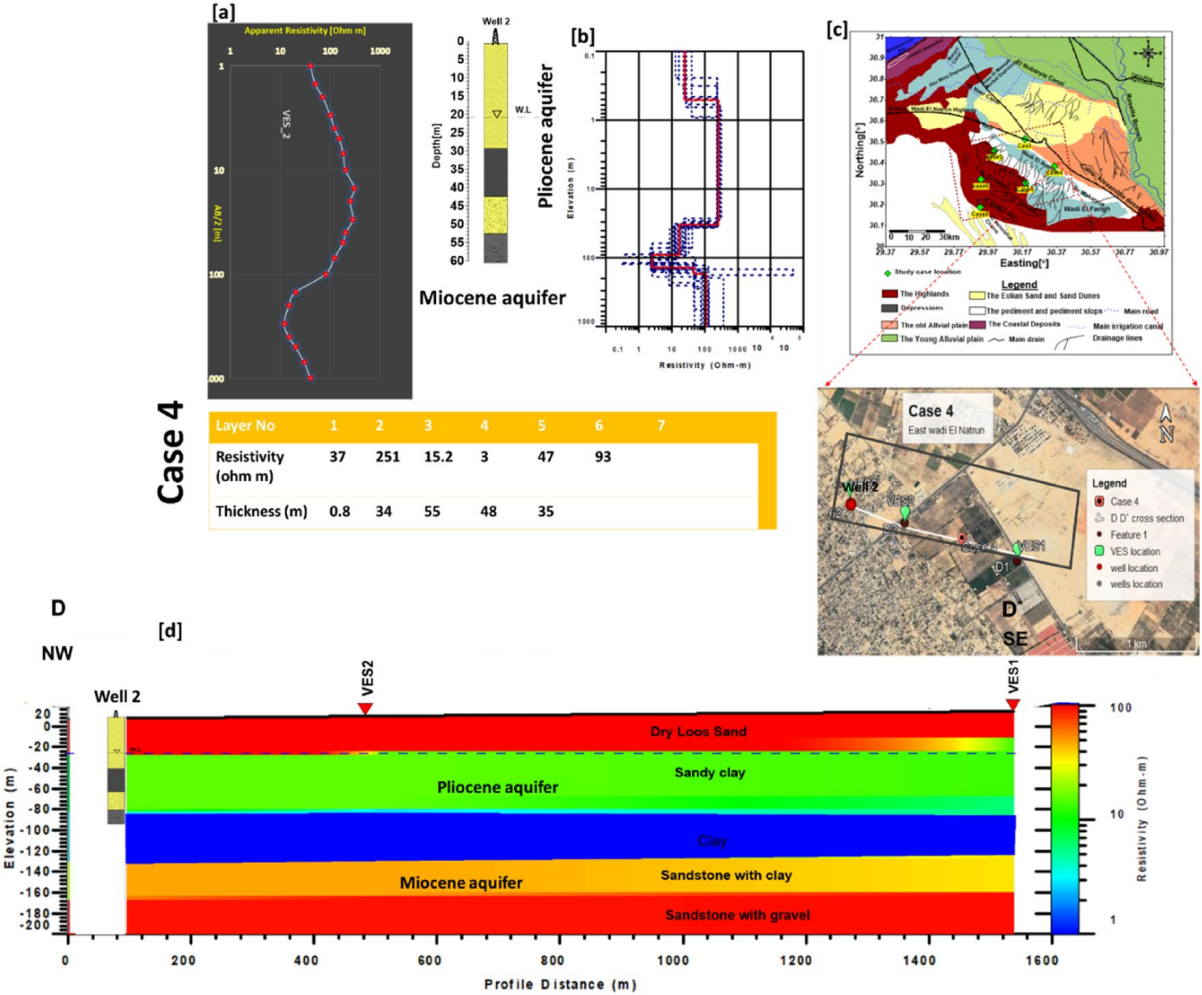
(**a**) VES2 as example for case 4 with Well 2, (**b**) inverted model with cumulative models, (**c**) the location of D–D′ cross section in the study area, (**d**) inverted resistivity cross section DD′ in the Fourth study area.

Is located on a farm to the East of Wadi El Natrun, specifically in the Nile Delta's south-west corner. This area is experiencing significant salinity and a drop in water levels. The water level is between 15 and 17.5 m deep. The basic model for data calibration was created using Well 2. To show the facies variations in lateral dimension, a cross-section D- D' was created by collecting two VESes (v1, v2) in the NW–SE direction. Figure 8 shows that, in addition to the weathered layer, the subsurface succession consists of four major units. The topmost layer has a relative resistivity value ranging from 20 to 35 Ωm and a thickness range from 0.5 to 8 m. The first layer has a resistivity range of 251–385 Ωm and a thickness range of 30–35 m. This layer exists because the drilling well (well 2) is made up of dry sand and gravel. The resistivity values drop with depth to 12–15 Ωm (second layer) at a thickness of 50–55 m. This layer might be made of sand clay with clay layers interbedded. This layer terminated at a depth ranging between 85 and 90 m, and it might have been the first aquifer (Pliocene aquifer). The inverted models were pointed out to a thickness of 45–55 m of low resistivity value, 5 Ωm (clay layer) at a depth of 85 m along cross section D-D` (Third layer). Subsequently, along the D–D′ cross section, the fourth layer, or the last layer, recoded a comparatively high resistivity of 30 Ωm when compared to the first aquifer (second layer). This layer, which didn't reach its full thickness, is regarded the second aquifer (Miocene Aquifer) of sands and gravel with clay lenes. It is found at a depth of 135–140 m.

### Case 5

**Figure 9:**
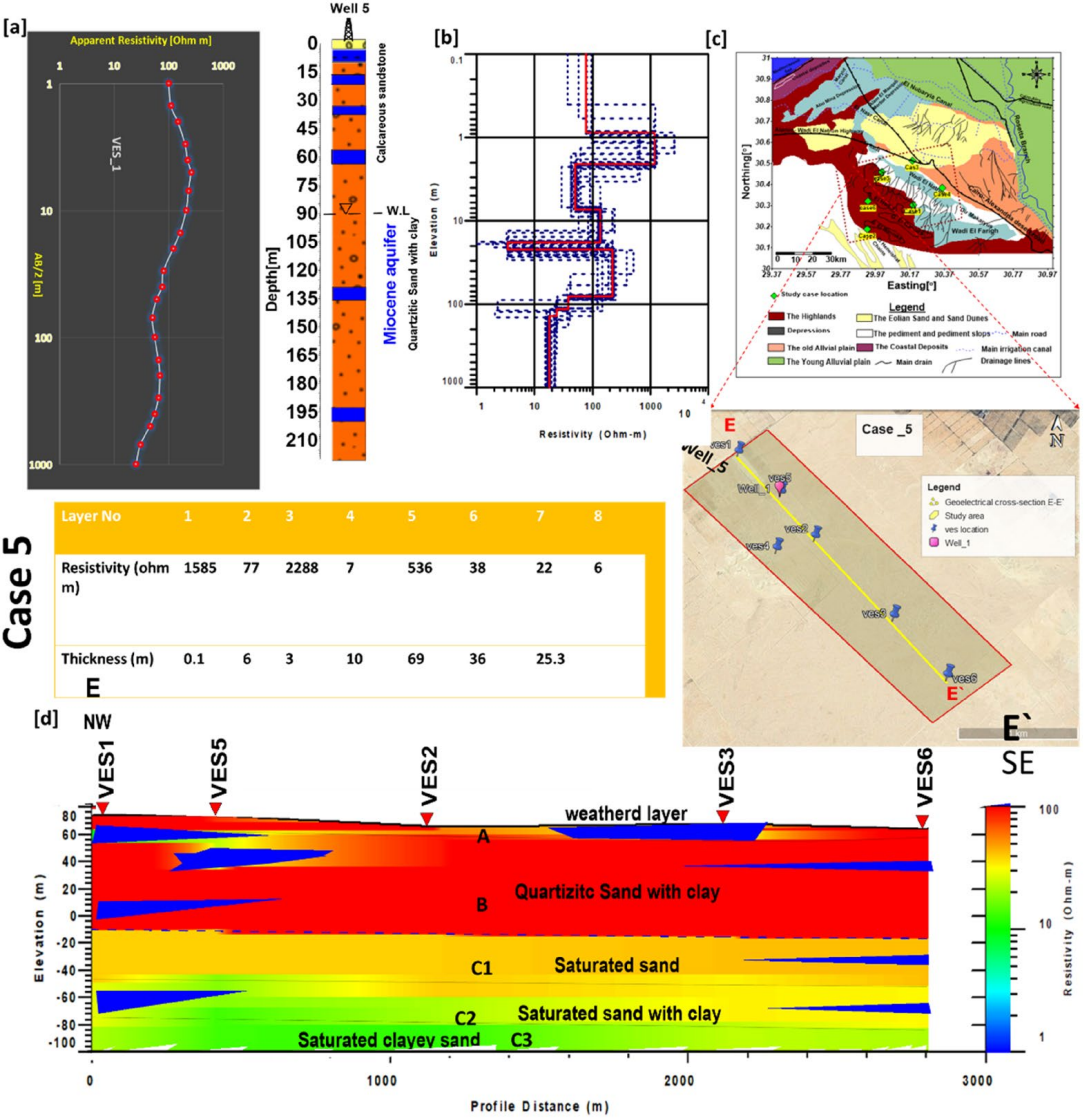
(**a**) VES5 as example for case 5 with Well 5, (**b**) inverted model with cumulative models, (**c**) the location of E–E′ cross section in the study area, (**d**) inverted resistivity cross section E–E′ in the fives study area.

This case is at the southern section of the Wadi El Natrun-El Alamein Road (Fig. 9). This region is 4200 m long and 150 m wide, parallel to the El Natrun-El Alamein Desert Road. Water level is recorded at 82 m due to the accessible drilling well inside the study case, and the water bearing is associated to the Pliocene aquifer, which is formed of sand, sandy clay, and coarse sand with clay interbed intercalation. Cross-section E-E' was created by collecting 6 VESes (v1, v2, v3, v4, v5, and v6) in the NE-SW direction to show the facies variations in both lateral and vertical dimensions. According to the findings, the subsurface succession is made up of three major units*.* The first layer (Unit A) has a relative resistivity of 9–1700 Ωm and a thickness of 2.1–6 m. This layer is characterized by a large range of resistivity values, which is related to facies alterations (changes from clay to sandy clay to sand). The second layer (Unit B) is considered unsaturated due to resistivity values ranging between 14 and 960 Ωm and a thick layer (80–98 m).

As illustrated in Fig. 9 along cross-section E–E′, the low resistivity value of 14 Ωm shows the intercalation of clay layers with sand and gravel beds. The third layer (Unit C) is classified as the saturated zone, and it is separated into three subunits (C1, C2, and C3) based on resistivity values, which indicate water quality (decrease with depth). Unit C1 is the best waterbearing layer (sandstone and sand deposits), with thicknesses ranging from 75 to 80 m and resistivity values ranging from 25 to 38 Ωm. Otherwise, the other subunits C2 and C3 are made up of sand deposits with clay bed intercalation, as the resistivity values in unit C2 decline from 16 to13 Ωm and from 12 to 9 m in subunit C3. The thickness of subunit C2 varies between 36 and 40 m. According to geochemical study, the salinity of the accessible water wells ranges between 3500 and 4100 ppm, which is corroborated by the results of decreasing resistivity values of saturated layers, particularly at subunits C2 and C3, where the clay content increases with depth.

### Case 6

**Figure 10:**
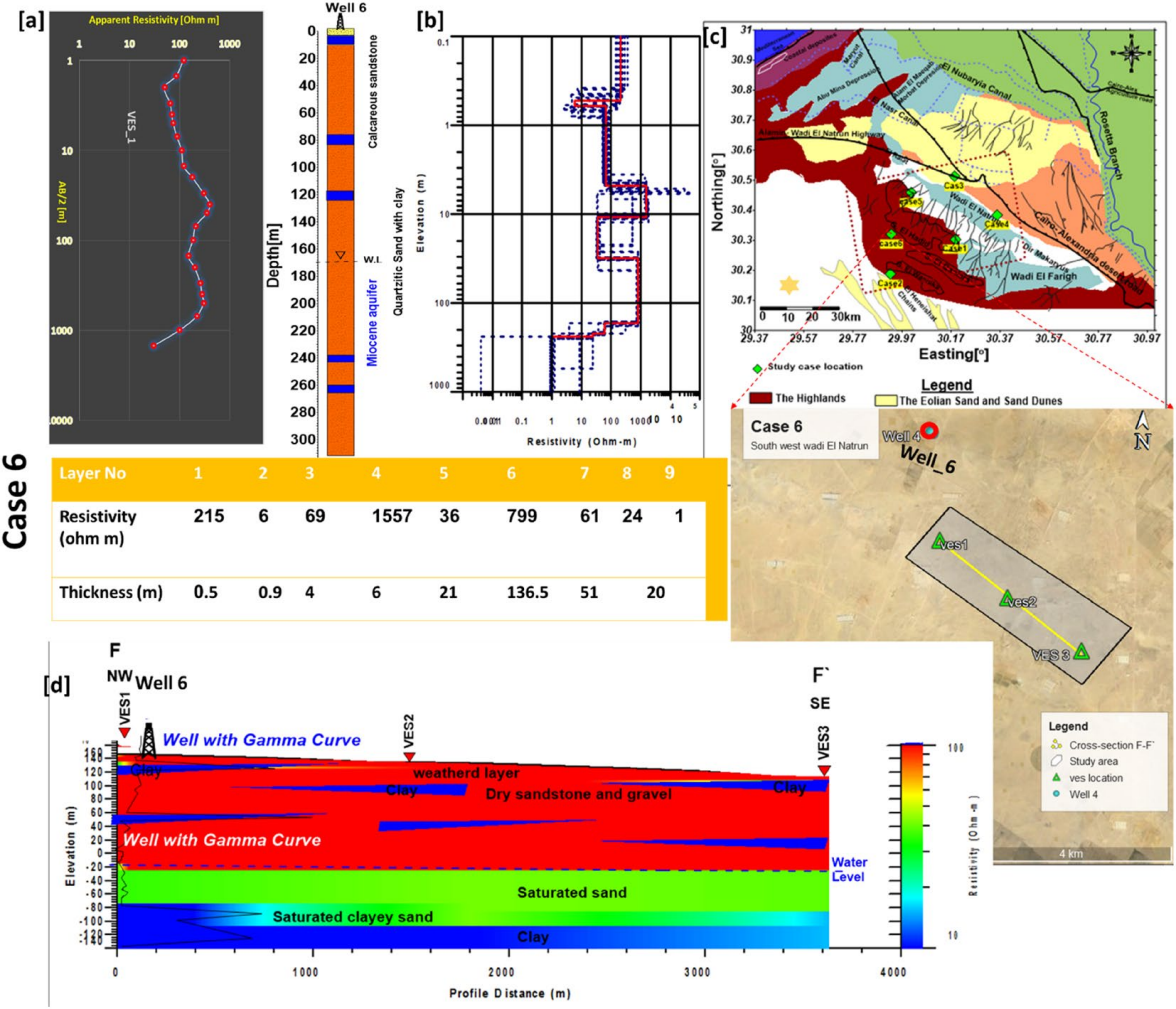
(**a**) VES1 as example for case 6 with Well 6, (**b**) inverted model with cumulative models, (**c**) the location of F–F′ cross section in the study area, (**d**) inverted resistivity cross section F–F′ in the sixth study area.

This case occurred southwest of Wadi El Natrun. The elevation at this site is between 140 and 150 m above sea level, according to the digital elevation map [DEM] (Fig. 2b). The waterbearing layer (Miocene aquifer) is located at great depths due to the nearby water well (Well 6). (165–180 m). The initial model was based on Well 6. The F-F cross section was designed to connect the three VESes (V1, V2, and V3) and identify changes in the facies in both vertical and lateral directions (Fig. 10). The NW–SE F–F′ cross-section is made up of four main units that are covered by a weathered layer of fine sand.

The first layer is made up of three dry layers of sand and gravel with resistivity values ranging from 70 to 1400 Ωm and a thickness ranging from 5 to 25 m. Furthermore, the second layer is dry and has resistivity values ranging from 50 to 2000 Ωm, while its thickness is measured at 125–150 m. This layer began at a depth of 25 m and extended at a depth of 165–180 m, and it is the unsaturated zone that overlies the Miocene aquifer at this location. The third layer is the saturated zone (waterbearing layer), which has a resistivity of less than 50 Ωm, a thickness of 50–65 m, and a depth of 220–230 m. Because of the low resistivity value (15 Ωm), the last layer (fourth layer) is classified as clay or clayey sand.

**Figure 11:**
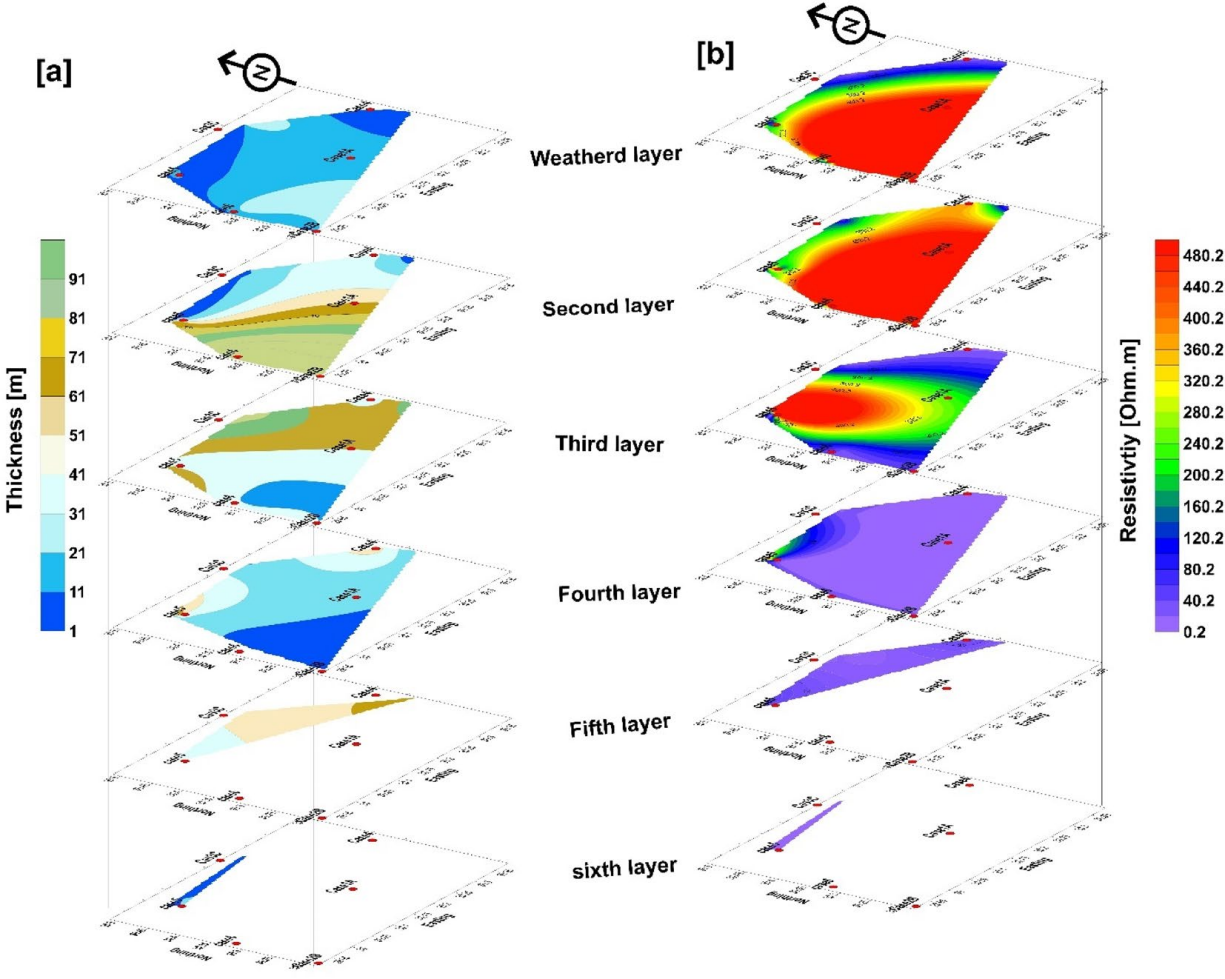
3D view contour maps. (**a**) Isopach map in (m), (**b**) Iso resistivity map in Ohm m for the all-case studies.

Figure 11a,b highlight the key findings from the 1D VES interpretation. The results are shown in 3D, with 4–6 primary layers under the case studies. The distribution of layer thicknesses is shown in Fig. 11a, whereas the distribution of resistivity values of layers is shown in Fig. 11b. In this study, the groundwater potentiality is primarily determined by parameters such as porosity, hydraulic conductivity, permeability, and clay content. The second layer is considered the promising waterbearing layer in cases 3, 4, and 1 which is related to Pleistocene and Pliocene aquifers of sediments rich in clay content, where layer 3 exhibits low resistivity values in north and east directions, but the thickness increases for the three cases. Cases 2, 5, and 6, on the other hand, are associated with the Miocene aquifer of sand and gravel deposits with clay lenses, as illustrated in Fig. 11, where the thickness of the clay layers decreases in the west and southwest directions. Based on the evidence of the geoelectrical resistivity value ((50 Ωm) of the waterbearing layer, the potentiality of groundwater in these localities is considered the best in the Wadi El Natrun districts.

### Morphometric analysis

**Figure 12:**
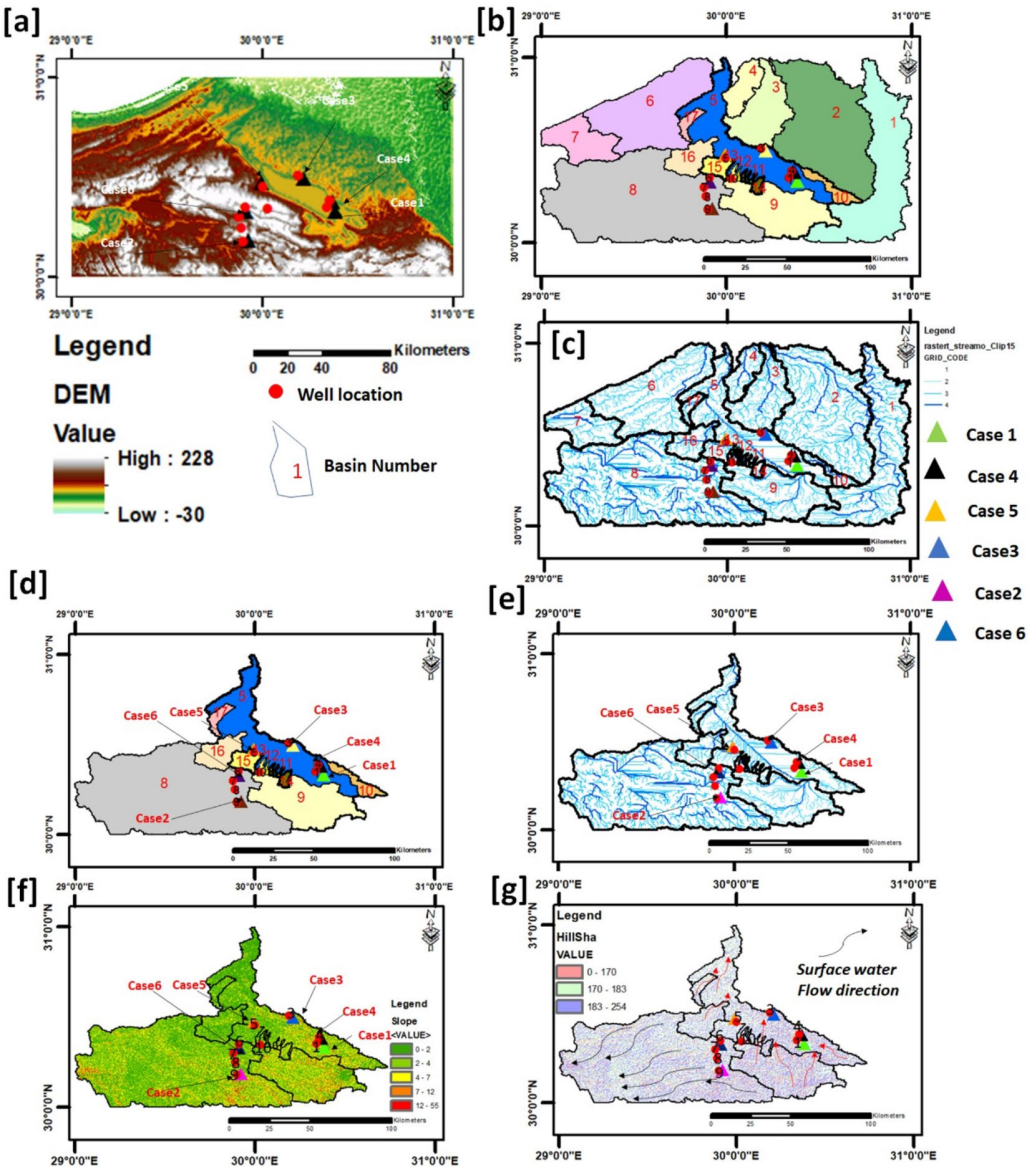
(**a**) Digital elevation map [DEM], (**b**) main basin and sub basins with number of each subbasins, (**c**) stream orders of the selected basins and subbasins, (**d**) area of interest (aoi) and its subbasins and attributes with the main stream orders, (**e**) slop map [0°–55°]. (**f**) Hill shade map with the main stream order of wadi El Natrun Basin_5 and basins B_8 to B_17, the pale blue arrows pointed out the directions of surface water to wadi El Natrun and its vicinities all over the studied cases.

As aforementioned, the intensive expansion of the reclamation process of the area under investigation over the last decades has been rapid and largely arbitrary, and the interpretation of the geoelectrical measurements of the six case studies addresses the study area's heterogeneous subsurface hydrogeological setting. The prior surface morphometric study would represent the different surface settings of the case studies and give insight into the continuous degradation of groundwater settings through time. ArcMap 10.4.1 software, used a digital elevation map [DEM] + 30 m of the study area to identify the main geomorphological units and to calculate the watershed for hydrogeological purposes and topographic analysis for Wadi El Natrun depression and its vicinities. Elevation and slope maps are shown in Fig. 12a,e, f. Wadi El Natrun Depression is oval in shape, roughly 50 km long, and 15–20 km wide. The overall area of the depression below sea level (0–23 m b.s.l.) is approximately 50,000 hectares. Because of its closeness and low level, the seepage from the Nile stream is the source of groundwater in Wadi El-Natrun, which confirms the hypothesis of^34^. Wadi El Natrun region is classified as severely dry, with mean annual rainfall, evaporation, and temperature of 41.4 mm, 114.3 mm, and 21 °C, respectively (Egyptian Metrological Authority, 1996). The research region has three main basins (B 1 (Nile Valley), B 5) Wadi El Natrun, and B 9) Wadi El Farigh, as well as 10 subbasins (B 2, B 3, B 4, and B 6). B 7, B 10, B 15, B 16, and Basin 17) as well as a few minor drainages (b11, b12, b13, and b14) that drain into the El Natrun basin (Fig. 12b,c).

As shown in Table 1, the area is divided into two sections: the area of interest (aoi) and the area out of interest (auoi). Figure 12d depicts the aoi of the overlapping stream orders of Wadi El Natrun basin and its subbasins, which comprise the most study cases (cases 1, case 3, case 4, case 5), and basin 8, which contains cases 2 and 6. Basin remnants, on the other hand, are regarded as (auoi). The area's topographic slope emphasizes its significance in influencing surface water flow. The slope level analysis separated the research area into four levels: extremely gentle 4°, gentle 4°–7°, moderate 7°–12°, and strong 12°–55° (Fig. 12e). The majority of the research area is gentle.

**Table 1:** Summary of interest/out of interest investigated areas (basins).

Basin No.	1	2	3	4	5	6	7	8	9	10	11	12	13	14	15	16	17
Local Name/Area of interest(aoi)/Area of out of interest (auoi)	Nile Valley Basin (out of interest)	Out of interest	Out of interest	Out of interest	El Natrun Basin (aoi)	Out of interest	Out of interest	aoi	aoi	aoi	aoi	aoi	aoi	aoi	aoi	aoi	aoi

As a result of morphometric analysis, all study cases are located on a moderate slope, except cases 5 and 6, which are located on a gentle slope level (Fig. 12f). The surface runoff is slow in the nearly level slope area (0–4) degrees, allowing more time for rainwater to percolate and be considered a good groundwater potential zone, whereas the strong slope area (12–55) degrees facilitates high runoff, allowing less residence time for rainwater, resulting in comparatively less infiltration and poor groundwater potential. shade map is very important in this study, where it clarifies the elevation models overlapping the main basins as shown in Fig. 12f, which is the main direction for water flow in the aoi. The aoi drainage density network was depicted in Fig. 12c. During the rainy season, the density of the drainage network reveals its influence on basin refilling via surface runoff. Limited drainage density has been shown to occur more frequently in areas that are extremely resistive to highly permeable subsurface material under thick vegetative cover and when relief is low. High drainage density is caused by impermeable or weak underlying material, scarce vegetation, and hilly terrain. Cases 2 and 6 show coarse drainage textures due to low drainage density, whereas case1 shows fine drainage textures due to high drainage density. The drainage density is proportional to the amount of runoff in a given region, or, in other words, the quantity of relative precipitation. As a result, the lower the drainage density, the greater the probability of recharge or potential groundwater zone (Cases 2, 5, and 6). The drainage map is split into eight orders, as shown in Fig. 12c. Figure 12f displayed the direction of water flow in the investigated (aoi), the recharge of Wadi El Natrun is mainly coming from southeast and west while the second (aoi) (basin no.8) is mainly coming from east and southwest towards the west direction.

### Groundwater condition

**Figure 13:**
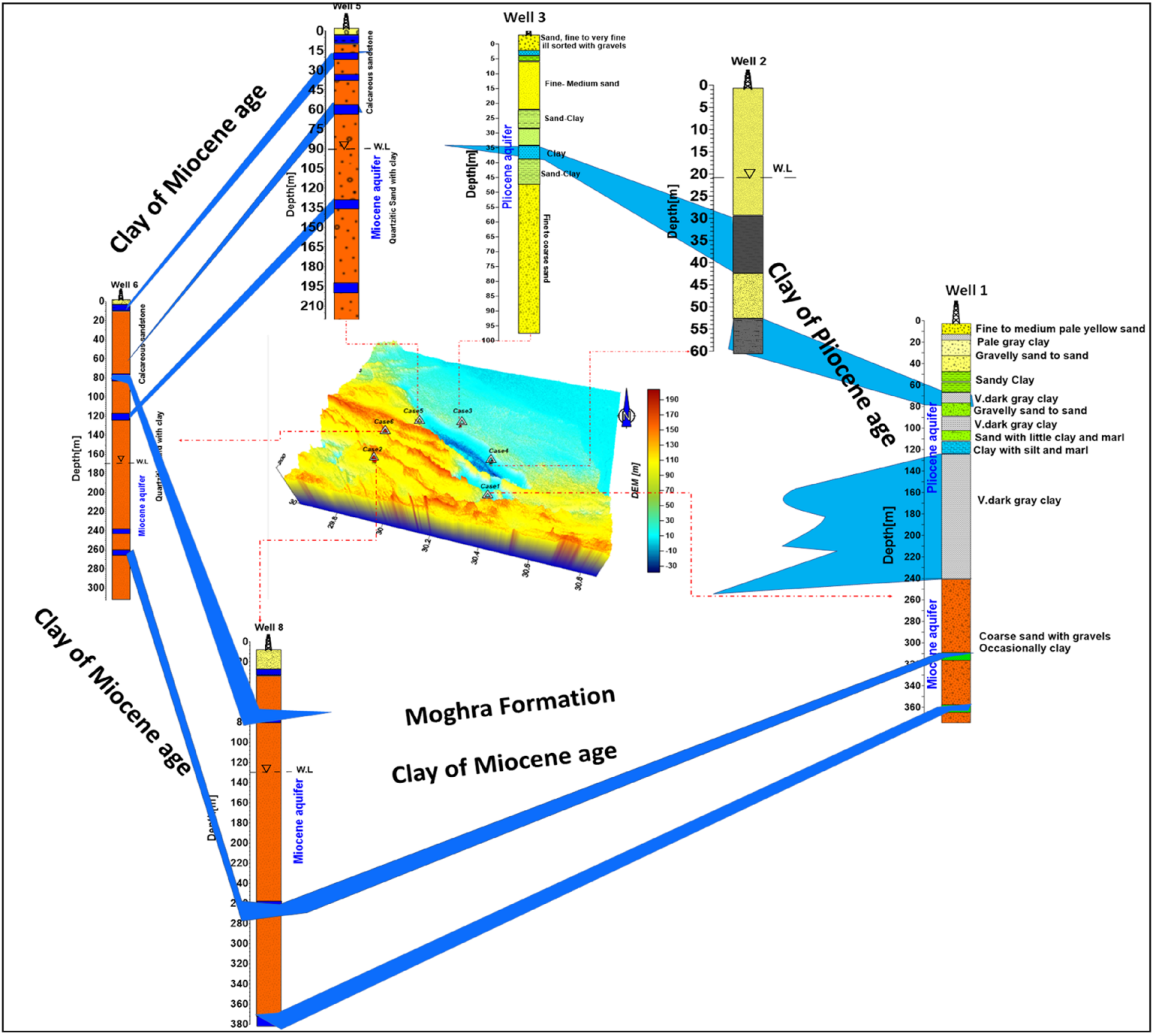
3D view of the initial models (drilled water wells) of resistivity inverted models of 1D VES with the expected clay layer/lens all over the studied cases.

**Figure 14:**
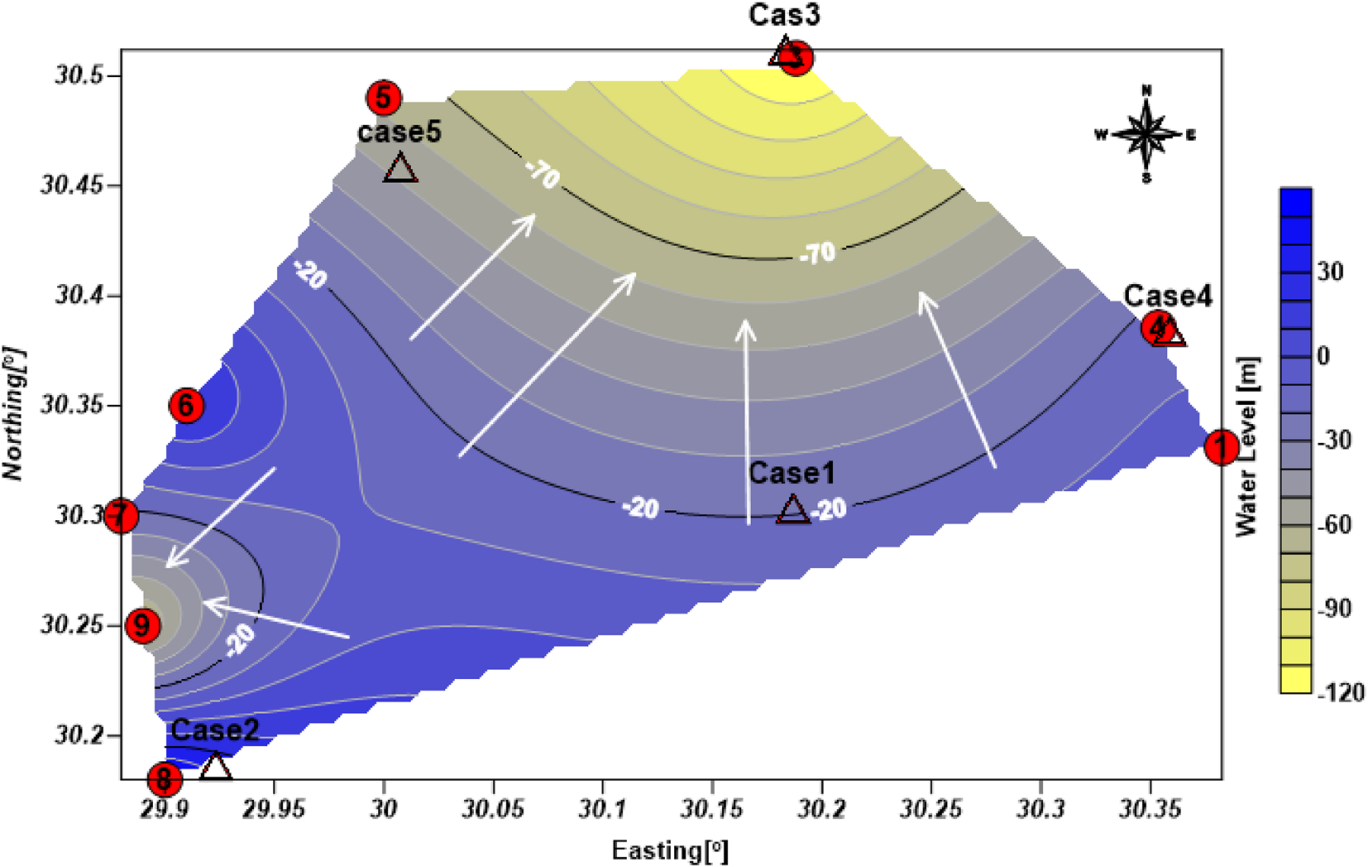
Simplified water level contour map of east and west Wadi El Natrun Depression, red circle represents available drilled wells with numbers and hollow triangle represents the location of case studies.

The current study found that the use of the resistivity method (1D VES) validates the subsurface hydrogeological setting of the studied cases on the phenomena of facies changes and the percentage of clay content with respect to aquifer ages, and is consistent with the findings of Salem^28^. The subsurface lithologic setting models of the six study cases utilized for the calibration of the interpretation of the geoelectrical data are depicted in Fig. 13. Cases 1, 3, and 4 are situated in the Pliocene age, which contains clay layers intercalated with sand lenses where, cases 2, 5, and 6, are found in a Miocene aquifer of sand and coarse gravel with a few clay lenses. Figure 14 depicts a simplified water level contour map for the cases studied. The results show two groundwater flow directions: one to the north and northwest of the study area, as determined by GIS analysis of DEM data in cases 1, 3, and 4, and one to the west in cases 2 and 6.

 T he results of this study support the results of^6,22^ that the principal waterbearing formations in the study area are Pleistocene, Pliocene, and Miocene sediments. In Cases 3 and 4, the Pleistocene aquifer occupies the northern portion of Wadi El Natrun basin. The Pleistocene aquifer's saturation thickness ranges from 57 m in the southwest to 334 m in the northeast. The steady increases in groundwater salinity over time are mostly the result of over-pumping and inadequate recharge. This aquifer's groundwater exists mostly under free water table conditions. In the east, the water depth exceeds 10 m. Groundwater flows usually from the east and north-west towards Wadi El Natrun depression, with isolated movements from the southeast and northeast. Such local groundwater migration trends are mostly related to over-exploitation of water to irrigate newly reclaimed lands. The Pliocene aquifer is primarily restricted to Wadi El Natrun depression and is primarily composed of clay with interbeds of waterbearing sandy layers, as in Case 1. The saturation thickness of the Pliocene aquifer ranges from around 132 m in the center of Wadi El Natrun basin to 51 m in the southeast. The Pliocene aquifer's clay content ranges from 84 to 12.5% (Table 2)^26–28^. Groundwater has been discovered at a depth of 30 m on the borders of Wadi El Natrun and is naturally flowing in the interior (Cases 1 and 4). Its productivity ranges from poor to moderate.

**Table 2:** Lithologic log analysis of some drilled water wells, around Wadi El Natrun Depression, after^28^.

Well nos	Ground elevation [m.a.s.l]	Depth to water [m]	Total depth [m]	Clay (%)	Sand (%)
1	− 20	− 15	60	–	–
3	37	21	101	–	–
4	6	160	66	–	–
5	60	84	220	29.5	70.5
6	152	169	313	13.1	86.9
7	132	153	266	20.3	79.7
8	114	129	380	–	–
9	103	121	270	12.2	97.8

The Miocene aquifer extends south and southwest of Wadi El Natrun and is mostly composed of coarse sands and clay lenses intercalated with water (Cases 2, 5, and 6). The saturated thickness of the Lower Miocene aquifer in the western portion exceeds 200 m^28^. The clay percentage of the Miocene saturated thickness ranges from 29.5% to zero %, based on drilling well data. The lateral facies changes are primarily responsible for the variation in hydraulic parameters laterally and vertically. The Miocene aquifer is bounded by NW–SE faults in the south Wadi El Natrun area, with its downthrown side to the east, where the Lower Miocene is uplifted opposite the Pliocene aquifer. As a result, this aquifer is hydraulically linked to the Pliocene aquifer underneath it^15^.

This study has found that morphometric analysis using GIS and the resistivity method at the different case studies show that the good potentiality zone is restricted to the west and southwest directions of the study area represented by layers 2 and 3 at cases 2, 4, 5, and 6, where the resistivity values of water bearing are relatively high and the choice of their locations is intuitive, as in cases 2 and 6. The other cases, on the other hand, are classed as poor potentiality zones since the estimated resistivity value is low due to clay deposits. Furthermore, their sites are deemed unsuitable due to their proximity to tiny attributers. As a result, the current research study demonstrates the significance of combining morphometrical analysis and geophysics techniques for such environmental problems, where groundwater is primarily controlled by geomorphological features and geological conditions, including lithology and geological structures.

## Conclusion

The geoelectrical resistivity approach was successfully used to determine the regional subsurface hydrogeological setting of six case studies located in the Wadi El Natrun and surroundings. When compared to other geophysical approaches, it was capable of discriminating between a wide range of hydro-geological facies. Using the resistivity method would allow for significant improvements in targeting local water supplies. The results of the resistivity measurements of 1DVES and 2DERI confirm that the groundwater setting via the different research cases is primarily determined by the phenomenon of facies alterations and the percentage of clay content with respect to aquifer ages. Cases 1, 3, and 4 are from the Pleistocene and Pliocene epochs and comprise clay layers intercalated with sand lenses where, Cases 2, 5, and 6nare situated in a Miocene aquifer of sand and coarse gravel with clay lenses. The Quaternary, Pliocene, and Miocene sediments are the most important water-bearing strata in the studied region. Finally, the integration of the results of the GIS analysis and resistivity method at the study cases revealed that the good potentiality zone is layer two and three at cases 2, 4, 5, and 6, where the evidence of resistivity values of water bearing are high and the choice of their location is favorable, where they are located at the main stream as case 2 and 6. On the other, the other cases are classified as poor potentiality zone as resistivity value recoded is low resistivity due to deposits rich with clay and their locations are not recommended, as they are located at small attributers . The authors highly recommended that 1D TEM sounding, 2DERT, and TDIP seem like better geophysical tools due to their high sensitivity to vertical and lateral changes in electrical conductivity and their ability to distinguish between clay and saline water, respectively, than the traditional DC VES method. Besides the soil and water analysis, the current study stresses the importance of performing detailed geomorphological analysis studies before the construction of new reclamation projects.

## Supplementary Information

Supplementary Information.
